# Advancing climate and health policies in Kenya: Insights from national policy stakeholders

**DOI:** 10.1016/j.joclim.2026.100750

**Published:** 2026-09-30

**Authors:** Richard Mulwa, Elvin Nyukuri, Kenneth Kigundu, Elly Musembi

**Affiliations:** a University of Nairobi, P.O. Box 30197, Nairobi, Kenya; b Chuka University, P.O. Box 109-60400, Chuka, Kenya

**Keywords:** Climate change, Health, Policies, Integration, Barriers, opportunities, Kenya

## Abstract

**Introduction:**

Climate change is increasingly recognized as one of the most critical determinants of global public health. The intersection of climate and health requires urgent attention if the impacts of climate change on health are to be mitigated*.* Kenya has been scaling efforts to address the challenges of the climate-health nexus, but the question of how to integrate climate change and health policy remains unanswered.

**Materials and methods:**

This study aimed to answer this question by conducting interviews with 21 high-level stakeholders working on climate change, health, and related policy areas in the months of April to July 2024. Data gathered from the interviews was supplemented by climate change and health literature.

**Results:**

Nearly all respondents agree that climate change and health policy should be explicitly integrated. This integration could enhance the use of financial resources, amplify co-benefits, and simplify the management of the two issues. Integration is, however, barred by the siloed nature of working and limited collaboration within these sectors, limited funding and data access, a shortage of skilled personnel, lack of political goodwill, and weak institutionalization of innovative ideas. To encourage integration, respondents identified interventions such as increased advocacy, public awareness campaigns, and international efforts focused on climate and health solutions. For improved collaboration, several suggestions including establishing cross-sector partnerships, conducting joint research, building capacity, and implementing holistic approaches like the One Health Model, were proposed.

**Interpretation:**

The findings from this study reveal that, although the national climate and health policy remain integrated to some degree, there exists several avenues and benefits of deepening the linkage. This study, therefore, provides a discussion on the potential implications of the findings for health and climate professionals, advocacy groups, researchers, government, the private sector, donors, and legislators.

## Highlights

• Integration of climate change and health policy could help address rising climate and health risks.
• Siloed nature of working and limited collaboration are key hinderances to integration.
• Integration can be enhanced through collaboration, improved policymaking, and capacity building.

## 1 Introduction

Climate change is making public health problems worse, increasing the risk of infectious diseases, food shortages, and mental health issues, especially for vulnerable groups [1,2]. Climate change impacts on health are becoming increasingly frequent and severe, prompting a growing prominence of health matters in climate change discussions [3]. Rising temperatures, severe weather events, and environmental deterioration necessitate the implementation of coordinated climate and health policies to attain Universal Health Coverage (UHC) and fulfil the Sustainable Development Goals [4,5]. However, extant literature demonstrates poor integration of climate change and health across countries [6], with climate-health related risks such as water-borne diseases and injuries from extreme weather, ignored or overlooked (for instance, malnutrition and mental health) in European countries [7]. In the United States (US), while notable efforts have been made to enforce regulations, particularly in developing heat and other early warning signs, limited emphasis has been given to communicating climate and health messaging, even within the trusted healthcare profession.

To promote integration of climate change and health, there have been global climate change discussions with a health focus such as the Health Day at Conference of the Parties (COP)28,^1^ COP28 Declaration on Climate and Health^2^ [8], and the 2024 G20 summit ministerial declaration on climate change and health.^3^ Discussions on integrating health into climate policies and programs featured prominently at the second African Climate Summit (ACS2) .^4^

In Kenya, climate change policymakers are aligning health issues with climate action by developing the Kenya Climate Change and Health Strategy, 2024–2029 [9]. While this is expected to harness synergies in addressing climate change and related health concerns, inadequate financing, human resource capacity, research, and technologies have been reported as some of the main hindrances to the implementation of the strategies or plans across countries. Others include competing priorities and inadequate multisectoral collaboration [10].

Building on existing literature and policy reports, this study provides insights from Kenya, a developing country where evidence remains scanty. The specific findings of this study have been published in a report [4]. In this article, however, we present the synthesized findings of the main report. Even though the original text has been rewritten in this article, some sentences from the report [4] could appear verbatim.

## 2 Methods

This study was part of a broader study on advancing climate change and health policies, focusing on six countries: Brazil, the United States (U.S.), the United Kingdom (UK), Germany and the Caribbean. These countries were chosen to represent low, middle and high-income country views, with the U.S team coordinating the research. All the country teams collaborated in developing the research protocols, including the research questions, interview guides, analysis plan and reporting layout, with inputs from the Global Climate and Health Alliance.

Prior to commencing the research, we obtained approval from the National Commission for Science, Technology and Innovation (NACOSTI), License No: NACOSTI/P/24/33,762. Key informant interviews were conducted in the months of April to July 2024, targeting actors in climate and health and related policy areas, including transport, agriculture, energy, economic planning and law. Target participants were purposively selected, with researchers using their professional networks and snowball sampling to recruit additional participants. Recruitment was done through email messages, and 21 participants were recruited. Fig. 1 provides a breakdown of recruited participants by disciplines and careers, respectively.

**Fig. 1:**
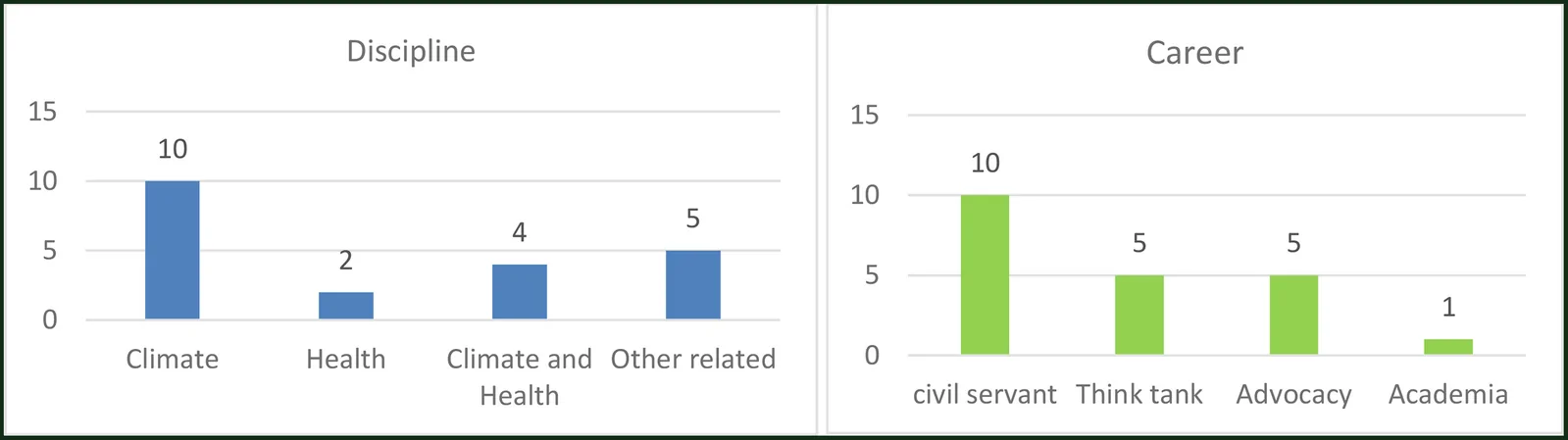
Disciplines and careers of respondents.

Interviews were conducted in English through Google Meet, Microsoft Teams, Zoom or in person. They were recorded after obtaining express consent from the respondents. Recorded interviews were later transcribed and analysed using a collectively determined inductive and deductive qualitative content analysis technique. Qualitative data collection was supplemented by literature survey in the areas of climate change and health. Data analysis was done systematically through identification of emerging codes in the data which were later developed into themes. These were later categorized into: current status of policy, optimal integration, barriers, opportunities and strategies.

## 3 Results

### 3.1 Current status of integration of climate and health policy

Most participants noted that climate and health policy in Kenya are yet to be clearly and fully integrated. For example, there is a notable absence of national strategies involving both climate and health, while the current climate change legislation and policy has not adequately addressed or integrated the health component. This notwithstanding, recent actions by the government appear to recognise the importance of this integration. These include environmentally sustainable healthcare programming, climate change trainings to health and non-health professions, and climate change and health vulnerability assessments. In addition, research linking climate and health has also begun, albeit at a slow pace, with findings helping shape health policies. Some participants also noted that the “One Health” approach is emerging as a good pathway to link climate and health. The approach is currently helping build community resilience in the Northern part of Kenya.

As these developments begin to take shape, the siloes between climate- and health-focused government institutions and the lack of an in-depth or explicit policy integration limit the full implementation. For instance, a respondent working in the Ministry of Health noted that though there exists a coordinating technical working group on climate and health bringing officials from different government institutions, the technical team has not achieved much, as it has been difficult to hold regular meetings due to poor coordination. There are, however, efforts to explicitly integrate climate and health policy, especially with the development of the Kenya Climate Change and Health Strategy, 2024–2029 [9].

### 3.2 Views about optimal integration

Nearly all the respondents were of the view that climate and health policy should be strongly integrated to leverage the synergies of solutions and benefits from climate and health interventions. The focal areas where climate and health policy should be integrated include agriculture, nutrition and food security, climate adaptation and resilience, emergency preparedness and disaster response, housing, and transport. Respondents also highlighted specific policy goals to incentivise climate and health policy integration such as enhanced funding, cross-sector collaborations, research, education and capacity building, increased awareness and strong advocacy. Other interventions include the development of national policy frameworks and strategies, setting national indicators and targets, and strengthening assessments of health and climate impacts of proposed national policies.

Some respondents noted that while assessments have previously been done, some have failed to consider both health and climate impacts. For instance, a respondent highlighted that the ongoing government driven affordable housing project has only considered climate impacts, effectively ignoring health impacts.

### 3.3 Barriers to optimal integration

Despite the limited success, optimal integration of climate and health policies has been hindered by;

*Siloed nature of working* among climate and health officials amplified the lack of integration. Also highlighted was the poor coordination in government sectors on climate and health issues. This was largely attributed to fear of sharing limited resources in joint activities across government institutions.

*Weak institutionalisation* of innovative ideas in government institutions making the ideas prone to risk of loss whenever there was change in administration or staff movement in the institutions. Further, bureaucracy and inefficiency in government institutions, and the lack of a dedicated unit working on climate and health matters in either the Ministry of Health or the Ministry of Environment, Climate Change and Forestry limited climate and health policy integration.

*Scarcity of resources* including inadequate funding, limited data on climate and health, and inadequate skilled personnel have compromised integration. Limited local funding of climate and health has paved way for increased donor-funding of many climate and health activities which prioritise the needs of the donor. Further, the focus given to climate and health policy integration in these donor-funded activities is very limited. The respondents also noted that most of the existing data and research output are on food, energy and water, with little attention given to climate and health policy. Even in instances where data and research output on climate and health are available, they are often difficult to access.

*Slow policy-making process and low uptake of evidence:* respondents indicated that policy-making process is very slow and characterised by weak implementation, partly due to a lack of non-binding commitments, a lack of political goodwill, and low uptake of evidence in policymaking. These continue to reinforce slow pace of integration.

*Limited training, poor awareness creation and advocacy:* respondents mentioned that there was a low level of understanding among policy makers, experts and the public on the link between climate change and health, largely due to limited training and awareness creation and advocacy programs. This is partly due to limited funding and failure to incorporate climate change and health issues in the education curriculum.

### 3.4 Opportunities to advance integration

The barriers highlighted above and others notwithstanding, opportunities for advancing integration of climate and health policies exist, and include;

*Advancement of climate and health policy integration at international and national levels.* This could be done through the Nationally Determined Contributions to the Paris Agreement and the Financing Locally-Led Climate Action (FLLoCA), an adaptation project with reach in the lowest administrative areas of the country and with potential for promoting integration at the national level.

*Creation of enabling conditions* that favor climate and health integration. These include the opportunity to leverage health benefits from climate policy and vice versa, increased public awareness on climate change impacts on health, enhanced global discussions on integration, and mounting public support for climate and health policy.

*Enhanced availability of resources* such as funding, data, and skilled personnel. Leveraging existing donor and local funding avenues could enhance integration if such funds allow for joint climate and health activities. Similarly, respondents mentioned the need for the country to take advantage of existing data and research outputs to integrate climate and health policy by making them easily available. In addition, Kenya can leverage on other resource opportunities such as the One Health or Planet Health initiatives.

*Development and implementation of strategies and policies:* Several practical opportunities that could be actualised in the near short term include the effective implementation of the Kenya Climate Change and Health Strategy (2024–2029) and the incorporation of health issues in national climate policies and vice versa. In addition, participants cited the need to incorporate climate and health matters in related policy areas such as water, energy, transport, and housing. These could be propelled by enhanced training, awareness and advocacy programs.

### 3.5 Strategies to advance integration

Alongside the opportunities for integration, there exist various strategies for actualising this integration such as;

*Strengthening Institutional Collaboration:* Continuous and structured collaboration among health institutions, climate agencies, and related sectors is essential for effective climate-health integration. To actualise this, respondents recommended institutionalisation of a dedicated climate and health unit domiciled either at the Ministry of Health or the Ministry of Environment, Climate Change and Forestry to coordinate integration. Such a unit would formalise intersectoral collaboration and safeguard continuity of innovative initiatives despite administrative transitions or staff turnover. The respondents further emphasized the need for stronger alignment between international, national and sub-national policy frameworks. Elevating climate and health discussions to the climate envoy’s office within the office of the President where high level climate negotiations are managed was also proposed to ensure political prioritisation and executive oversight.

*Streamlining the policy-making process:* Respondents advocated for the development of a robust and coherent strategy linking climate and health policies. Key recommendations include enhancing meaningful citizen participation, strengthening the use of scientific evidence in policy making, and simplifying legislative procedures to accelerate implementation. A greater involvement of climate and health experts alongside sustained political goodwill from national leadership will be critical.

*Enhancing research engagement:* the role of researchers in supporting climate and health policy should be expanded. This will be important for collecting and providing data and generating policy-relevant evidence particularly research quantifying the health costs of climate change. Emphasis should be on multi-disciplinary research to avoid the siloed approach to research.

*Strengthening advocacy and communication:* Strategic advocacy efforts should leverage social media platforms, mobilize citizen advocates, and organize civil society organisations into coordinated networks capable of influencing policy makers. Respondents identified strategies to enhance communication including messaging quantifying the health cost of climate change, unified messages highlighting the benefits of integration, the use of experts such as climate and health professionals to communicate and express climate change in terms of health to audiences.

*Capacity building* is needed particularly to enhance technical and analytical skills of government professionals working in climate and health. Policy makers should also receive targeted training to enhance their understanding of climate health linkages so they can help streamline policymaking processes.

*Promoting the One Health framework:* This approach offers a practical framework for integrating human, animal and environmental health concerns in the climate-health nexus. Its application has been demonstrated among pastoral communities in northern Kenya where climate related shocks disproportionately affect livestock dependent livelihoods. To supplement government and donor programs to enhance the resilience of these communities, the private sector has stepped in to provide insurance packages for livestock.

## 4 Discussion

It is becoming even more evident that health crisis from climate change has been increasing in recent decades [12,13]. Public health is expected to continuously play an essential role in climate planning and action as the co-benefits of health help underpin low carbon economy strategies, while safeguarding health [11]. This notwithstanding, health debate has been elusive in its influence on climate mitigation policy due to vested economic interests and structural political issues [14]. The views of respondents in this study are largely in line with this literature, showing limited climate and health policy integration [7,11,15]. The findings reveal that climate and health policy integration is constrained by structural, institutional and political barriers, yet significant opportunities and actionable strategies exist to overcome these limitations.

Among the notable barriers to integrating climate and health is the siloed nature of collaboration between climate and health professionals, a limited understanding of the impacts of climate change on health, and minimal coordination between the two disciplines [4,10]. There is also competition over limited financial and human resources which reinforces fragmentation. Further, absence of a dedicated climate and health unit limits continuity and accountability. Other barriers include inadequate funding, data and limited technical expertise. These barriers could be addressed by increased funding, capacity building, enhanced health messaging, improved coordination among climate, health, and other relevant actors and research and data support [8,11,15]. On its part the WHO at COP 2019 urged world leaders to abandon the siloed approach to addressing climate change and health. It stressed the importance of positioning health at the core of all climate negotiations, strategies, policies and action plans, to save lives and secure healthier futures for present and future generations [8].

Despite these constraints, opportunities for advancing integration are evident. At the international level, embedding health considerations within the Nationally Determined Contributions (NDCs) offers a formal mechanism for policy alignment, enhanced accountability and access to climate finance. National initiatives such as FLLoCA can create pathways for translating national commitments into localised adaptation interventions, thus strengthening vertical integration. Increased public awareness of climate related health risks, global recognition of climate health discussions, and the growing demand for climate action create a conducive policy environment for enhanced integration. Resource mobilization also presents opportunities as existing funding streams from various donors can be leveraged to support integration. Similarly, use of existing data and research outputs can enhance evidence informed policy making. Finally, the One Health approach is very critical as it offers a framework for aligning the climate and health nexus. At the National level, effective implementation of the Kenya Climate change and health strategy 2024–2029 provides an immediate practical opportunity.

Addressing barriers while capitalizing on opportunities requires coordinated strategies. These strategies include strengthening institutional collaboration, streamlining policy making, enhancing research engagement, strengthening advocacy and communication, capacity building, and promoting one health framework. If these are addressed within one coherent governance framework, Kenya can advance a long lasting and effective climate and health policy integration pathway.

## 5 Conclusion

The findings of this study emphasise the need and opportunities to advance integration of health into climate policy. This, however, is not without various barriers that impede integration. This study implores all actors interested in good health outcomes within a healthy planet to consider the findings and adapt their strategies accordingly. These results have potential implications for different actors:

*The Executive Office of the President* to give more prominence to climate and health policy integration by elevating climate and health discussions to the climate envoy’s office. This is the office where high-level climate negotiations, including COPs, are housed. Elevating these discussions to this office will not only make them feature prominently in government programs but also attract donor support.

*The National Assembly of Kenya* can build on the results of this study to craft ambitious, feasible and binding legislation on climate and health policy. A climate and health lens can also be introduced into legislation on related areas including agriculture, transport, housing, water and energy. Further, with its key role in the budget-making process, the parliament can allocate more funds to climate and health policy integration. Moreover, parliament can, through its oversight role, monitor progress in the implementation of climate and health policy.

*Public servants* can ensure full implementation of the climate and health policies. They can push for more collaborations across national and sub-national levels and ensure effective mainstreaming of climate and health issues in their workplaces by embracing and training on the health impacts of climate change.

*Climate professionals* can push for collaborations with health professionals to build lasting relationships and leverage the benefits of synergies. To build strong relationships, climate professions need to strongly highlight the relevance of health in climate policy. Additionally, these officials can push for broadening climate curricula to include health issues.

*Health professionals* can also push for collaboration with climate professionals. They can also play a significant role in pushing for integration by effectively communicating and quantifying the health impacts of climate change, particularly extreme weather events. They can further push for the integration of climate issues into the health curricula.

*The research* community can collect data and generate relevant information needed to support integration and make existing resources accessible to policymakers. Further, they can support key research needs, including quantifying the health impacts of climate change, the economic costs of health problems related to climate change and the benefits of climate and health policy integration.

*The donor community and private sector* can play a crucial role in addressing inadequate funding, a barrier that features prominently in the actualisation of the climate and health policy. The private sector should be invited into the conversations and encouraged to set up a special fund to support the integration. Public-private partnerships could also be useful in financing high-resource-demanding programs.

*Advocacy groups* can mobilise citizens to push policymakers to prioritise climate and health policy legislation. They can also use effective communication strategies to create public awareness of the health costs of climate change and the benefits of climate and health policy integration.

## 6 Limitations

It is believed that the views in the study are reflective of climate and/or health actors in Kenya, although it was difficult to recruit legislators and high-level civil servants, thus the potential self-selection bias. Despite this limitation, views of sampled actors in climate and health spaces are to a large extent representative of opinions of the broader spectrum of actors, even those not adequately represented. Further, the study was mainly limited to the national level stakeholders, even though policymaking at the sub-national and international levels is crucial to climate and health policy integration. Future studies should include sub-national actors, legislators and other stakeholders not accounted for in this study. Further, more rigorous study designs could be employed to overcome any biases. Finally, respondents could have potentially, intentionally or unintentionally, aligned their views with those of the interviewer’s, thus introducing more response bias.

## Data sharing statement

Due to the sensitive nature of some of our interview questions, the data collected for the study are confidential and will not be archived publicly.

## CRediT authorship contribution statement

**Richard Mulwa:** Writing – review & editing, Writing – original draft, Supervision, Project administration, Methodology, Investigation, Conceptualization. **Elvin Nyukuri:** Writing – review & editing, Writing – original draft, Methodology, Investigation, Formal analysis. **Kenneth Kigundu:** Writing – original draft, Methodology, Formal analysis, Data curation. **Elly Musembi:** Writing – original draft, Methodology, Formal analysis, Data curation.

## Declaration of competing interest

The authors declare that they have no known competing financial interests or personal relationships that could have appeared to influence the work reported in this paper.

## Footnotes

1 https://www.who.int/news-room/events/detail/2023/12/03/default-calendar/cop28-health-day

2 https://www.who.int/publications/m/item/cop28-uae-declaration-on-climate-and-health

3 https://www.g20.utoronto.ca/2024/241031-health-declaration-climate-equity.html

4 https://climatehealthevidence.org/updates/africa-climate-summit-pathways-integrating-health-africas-climate-change-adaptation
